# Association Between Obstructive Sleep Apnea-Hypopnea Syndrome and Outcomes in Patients With Myocardial Infarction in the Absence of Obstructive Coronary Artery Disease

**DOI:** 10.3389/fcvm.2020.573819

**Published:** 2020-10-23

**Authors:** Chao-Jie He, Lin-Feng Cao, Chun-Yan Zhu, Xiao-Ce Dai, Yue-Yan Yu, Yu-Juan Zhu, Chang-Lin Zhai, Gang Qian, Hui-Lin Hu

**Affiliations:** ^1^Jiaxing Key Laboratory of Arteriosclerotic Diseases, Department of Cardiology, The First Hospital of Jiaxing, The Affiliated Hospital of Jiaxing University, Jiaxing Institute of Arteriosclerotic Diseases, Jiaxing, China; ^2^Department of Respiration, The First Hospital of Jiaxing, The First Affiliated Hospital of Jiaxing University, Jiaxing, China; ^3^Department of Anesthesiology, The First Hospital of Jiaxing, The First Affiliated Hospital of Jiaxing University, Jiaxing, China

**Keywords:** obstructive sleep apnea-hypopnea syndrome, coronary artery disease, outcome, mortality, major adverse cardiac and cerebral event (MACCE)

## Abstract

**Background and Aims:** Myocardial infarction in the absence of obstructive coronary artery disease (MINOCA) occurs in 5–10% of all patients with acute myocardial infarction. Obstructive sleep apnea-hypopnea syndrome (OSAHS) is linked to increased cardiovascular morbidity and mortality, but the relationship of OSAHS and outcomes in patients with MINOCA remains unknown. We aimed to evaluate the association between OSAHS and clinical outcomes in patients with MINOCA.

**Methods:** Between January 2015 and December 2016, we carried out a consecutive cohort study of 583 patients with MINOCA and followed them up for 3 years. An apnea-hypopnea index of ≥ 15 events per hour recorded by polysomnography was defined as the diagnostic criterion for OSAHS. The primary end point was all-cause mortality, and the second end point was major adverse cardiovascular or cerebrovascular events (MACCE), a composite of cardiac death, non-fatal myocardial infarction, heart failure, cardiovascular-related rehospitalization, and stroke.

**Results:** All-cause mortality happened in 69 patients and MACCE occurred in 113 patients during the 3-year follow-up. Kaplan–Meier survival curves indicated the significant relationship of OSAHS with all-cause mortality (log-rank *P* = 0.012) and MACCE (log-rank *P* = 0.002). Multivariate Cox regression analysis indicated OSAHS as an independent predictor of all-cause mortality and MACCE [adjusted hazard ratio: 1.706; 95% confidence interval (CI): 1.286–2.423; *P* = 0.008; and adjusted hazard ratio: 1.733; 95% CI: 1.201–2.389; *P* < 0.001; respectively], independent of age, sex, cardiovascular risk factors and discharge medications.

**Conclusions:** OSAHS is independently associated with increased risk of all-cause mortality and MACCE in patients with MINOCA. Intervention and treatment should be considered to alleviate OSAHS-associated risk.

## Introduction

Myocardial infarction in the absence of obstructive coronary artery disease (MINOCA) is a distinct entity with multiple causes defined by acute presentation of myocardial infarction with no remarkable stenosis of coronary artery on coronary angiography (stenosis <50%) (1–3). In June 2019, the American Heart Association (AHA) has systematically described recommendations for contemporary diagnosis and management of MINOCA, and they have broadened our horizon to understand this heterogeneous disease (4). More than 660,000 percutaneous coronary intervention (PCI) procedures were performed in China in 2017, and ~10% of emergency coronary angiography results showed the absence of evident obstructive coronary stenosis (5). Although the number of initial diagnosis of MINOCA is considerable owing to the huge population and demand for PCI, the clinical outcomes, and follow-up of MINOCA have received minimal attention with the result indicating a portion of individuals may not receive appropriate treatment (6, 7).

Sleep apnea-hypopnea syndrome (SAHS) is a syndrome of nocturnal respiratory interruptions resulting in snoring, sleep fragmentation, and daytime drowsiness, with a prevalence ranging from 5.7 to 17% in adults (8–10). Obstructive SAHS is the most common manifestation of sleep apnea and it is caused by nocturnal upper airway collapse with paradoxical thoracoabdominal movement (8). In several multi-center registries, obstructive sleep apnea was observed in 45.3–65.7% of patients with myocardial infarction or who underwent PCI (11, 12). Accumulating evidence has demonstrated that OSAHS is an emerging risk factor for arteriosclerosis, coronary heart disease, hypertension, stroke, cardiovascular rhythm, and heart failure (HF) (13–17). Numerous studies have shown that continuous positive airway pressure (CPAP) therapy could retard the progression of coronary artery disease (CAD) and decrease the rate of occurrence of cardiovascular mortality, stroke, and non-fatal myocardial infarction (18–21).

To our best knowledge, no published data are currently available concerning the prevalence of OSAHS in patients with MINOCA. In addition, the association between OSAHS and clinical outcomes has not been determined in this population. This study aimed to investigate the prevalence of OSAHS in patients with MINOCA and to evaluate the relationship between OSAHS and clinical outcomes in patients with MINOCA.

## Methods

### Study Design and Population

Our study complied with the Declaration of Helsinki and was approved by the Ethics Committee (The First Affiliated Hospital of Jiaxing University, Jiaxing, China). All participants provided their informed written consent before being enrolled in the present study.

A total of 583 patients who had been referred to First Affiliated Hospital of Jiaxing University for emergency coronary angiography and diagnosed with MINOCA were included consecutively in the present study between January 2015 and December 2016. Jiaxing University Hospital has a Chest Pain Center that covers a 4,000 sq. km area and serves about six million residents. MINOCA was diagnosed with the following criteria in line with a position paper of European Society on cardiology: (1) the diagnostic criteria involving the Fourth Universal Definition of Myocardial Infarction criteria; (2) coronary angiography with no artery lesions ≥ 50% in any infarct-related artery; (3) no other clinically overt specific cause that can explain acute presentation (22). The exclusion criteria were extremely strict in the present study: (1) mimic myocardial infarction, such as sepsis, pulmonary embolism, cardiac contusion, overlooked obstructive CAD, coronary emboli/thrombus, Takotsubo syndrome, and myocarditis (4); (2) cognitive disorder or mental confusion; (3) known OSAHS on CPAP therapy; (4) patients' refusal to give a written informed consent. The patients were divided in two groups, namely, the OSAHS, and non-OSAHS groups, in accordance with the evaluation of OSAHS.

### Data Collection

We used inpatient record system to collect the data on demography, laboratory, electrocardiogram, echocardiography, coronary angiography, and discharge medication. Data collectors were blinded to the study group assignment. After discharge, all patients were followed-up for 3 years by physicians in First Affiliated Hospital of Jiaxing University. Follow-up was performed by outpatient clinic visit, patient record system, telephone contact or instant messaging (WeChat). In case of failure to contact the patients, we used their family members' contact information and asked patients to visit the outpatient clinic annually. All individuals were advised to contact us immediately if they experience angina or uncomfortable chest stuffiness.

### Diagnosis of OSAHS

All enrolled patients were required to complete the Berlin Questionnaire (23). The Chinese version of Berlin Questionnaire is a convenient and inexpensive self-assessment method, which consists of 10 questions in three categories. The questionnaire assesses the presence of sleep apnea by persistent symptoms of snoring, the severity of daytime sleepiness, and a history of hypertension or a body mass index (BMI) > 30 kg/m^2^. A positive score in at least two out of three categories indicates a high risk of OSAHS (23).

All individuals were asked to monitor their overnight sleep by using a polysomnography device (SOMNOmedics GmbH & Co. KG, Germany) with the assistance of pneumology department regardless of their results on the Berlin Questionnaire. Polysomnography is the golden standard for obstructive sleep apnea diagnosis. The apnea-hypopnea index (AHI) was quantified as the average number of apnea and/or hypopnea per hour of the total recorded time. Apnea was defined by a 90% or greater reduction in airflow lasting at least 10 s. Hypopnea was defined as a 30% or greater drop in respiratory airflow lasting ≥ 10 s accompanied with an oxygen desaturation ≥ 4% (24). Obstructive sleep apnea was defined as the absence of airflow in the presence of paradoxical thoracoabdominal movement. An AHI of > or = 15 events/h was considered prospectively as the criterion for OSAHS (AHI values of 5–15, 15–30 and > 30 indicate mild, moderate and severe symptoms, respectively) (24).

### Primary and Secondary End Points

The primary end point in the present study was all-cause mortality. The secondary end point was a major adverse cardiovascular or cerebrovascular events (MACCE), defined as a composite of cardiac death, non-fatal myocardial infarction, HF. cardiovascular-related rehospitalization, and stroke. Cardiac death refers to death with evidence of acute coronary syndrome, cardiac arrhythmia or congestive HF. The diagnosis of stroke was performed in line with the AHA guidelines for the scientific statement of stroke (25). The definition of HF was determined in accordance with the current guidelines published by European Society of Cardiology (26). Cardiovascular-related rehospitalization means any cardiovascular causes for readmission. Mortality and MACCE were first verified by searching the patient record system by means of HaiTai software. Death certificates were obtained for any patients who died during the follow-up period if available. All individuals were then contacted by telephone or instant messaging (WeChat) for assessment of end points. End point confirmations were conducted by two physicians without any knowledge of diagnosis of OSAHS.

### Statistical Analysis

Data were analyzed with the SPSS version 19.0 software (IBM, SPSS Inc., Chicago, IL). Continuous variables were presented as the mean and standard deviation and categorical variables were expressed as frequencies and percentages. Differences between means were analyzed with Student *t-*test. Differences between proportions were assessed with Chi-square test or Fisher's exact test as appropriate. Kaplan–Meier curves of the survival function were calculated for patients with OSAHS and non-OSAHS, and the two groups were compared with the log-rank test. Multiple Cox proportional hazards model was used to adjust for potential confounders using stepwise regression (modification of the forward selection method) with *P* < 0.10 in the univariate analysis. The proportional hazards assumption was evaluated for Cox regression. Results of Cox regression are presented as hazard ratio (HR) with 95% confidence interval (CI). A two-tailed *P* < 0.05 was considered statistically significant.

## Results

### Baseline Characteristics of Patients

**Figure 1:**
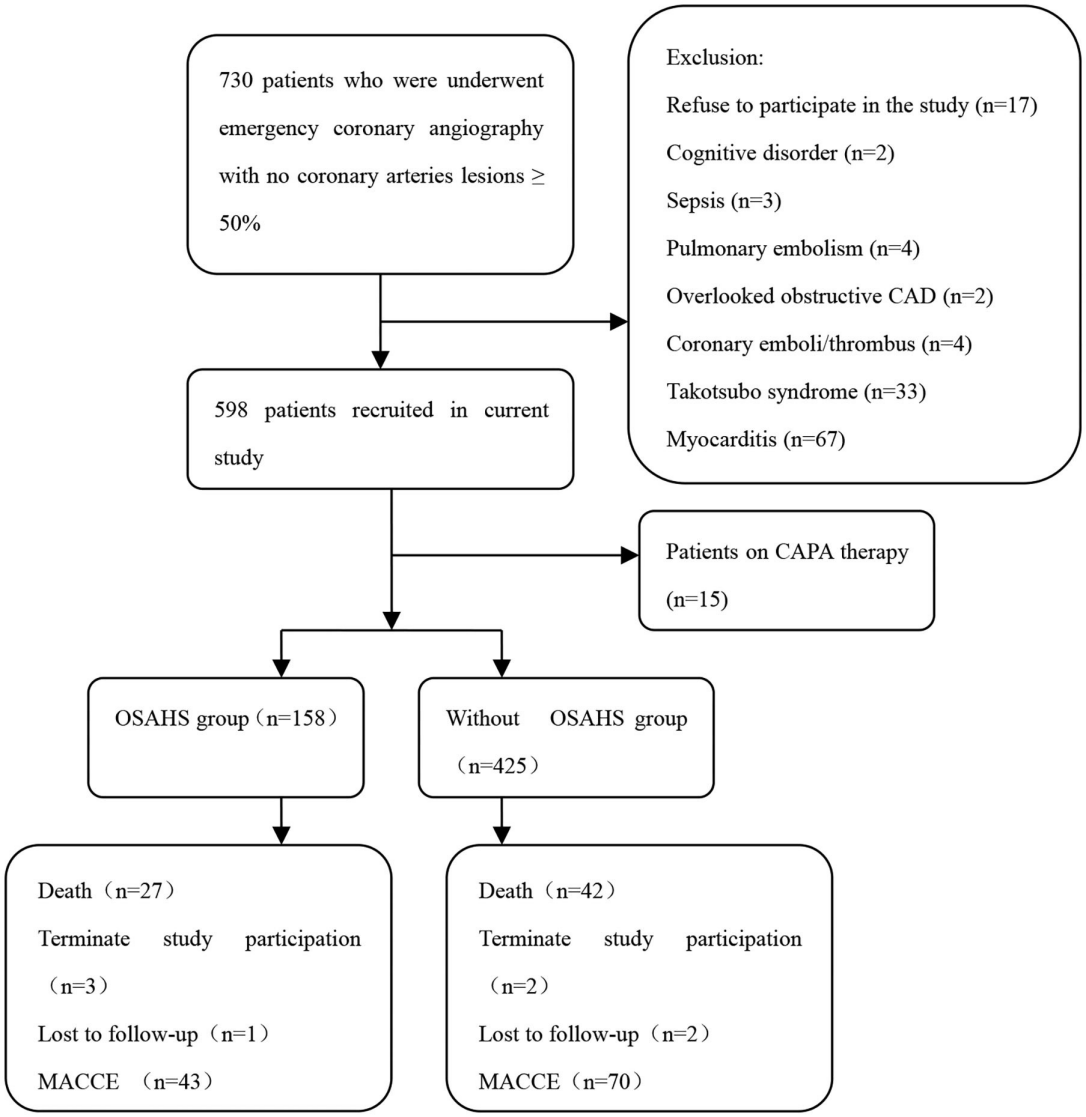
Flowchart of the selection process and dropouts of the current study. CAD, coronary artery disease; CPAP, continuous positive airway pressure; OSAHS, obstructive sleep apnea-hypopnea syndrome; MACCE, major adverse cardiac or cerebrovascular events.

The study objects included 730 patients with acute myocardial infarction and non-obstructive coronary arteries. A total of 147 patients were excluded from the present trial: 2 with cognitive disorder, 3 with sepsis, 4 with pulmonary embolism, 2 with overlooked obstructive CAD, 4 with coronary emboli/thrombus, 33 with Takotsubo syndrome, 67 with myocarditis, 15 patients on CPAP therapy, and 17 patients who refused to participate in the study. This prospective cohort study recruited 583 patients with MINOCA (Figure 1), of which 8 individuals terminated study participation or were lost to follow-up. The Berlin Questionnaires of 237 patients have indicated a high risk of sleep apnea and/or hypopnea, and 158 patients were diagnosed with OSAHS by polysomnography, with a prevalence of 27.1%. Whereas, only 70.9% of the patients in the OSAHS group were classified as high risk by the Berlin Questionnaire. Patient baseline characteristics are listed in Table 1. Compared with the control group, OSAHS patients had a higher proportion of males and a history of smoking but without further significant differences regarding age, BMI, cardiovascular risk factors, medications at discharge, laboratory, electrocardiogram, echocardiography, and coronary angiography data. Table 2 shows the sleep results from OSAHS and non-OSAHS groups. Of 158 OSAHS individuals, 84 presented moderate symptoms, and 74 exhibited severe ones. The mean AHI values were 29.0 ± 12.2 and 5.3 ± 4.2 for OSAHS and non-OSAHS groups, respectively. The mean lowest SpO2 values during apnea were 82.7 ± 7.9 and 91.3 ± 4.1, respectively. Fifteen patients with moderate or severe OSAHS agreed to receive CPAP treatment. The present study was to investigate the association between untreated OSHAS and clinical outcomes in patients with MINOCA, patients who had received CPAP therapy for more than 1 month during the study were excluded from analysis.

**Table 1:** Baseline characteristics of the study population with or without OSAHS.

**Characteristics**	**With OSAHS**	**Without OSAHS**	***P*-value**
	**(*n =* 158)**	**(*n =* 425)**	
Demographics			
Age, mean ± SD, y	64.5 ± 12.9	63.4 ± 12.7	0.158
Female, n (%)	62 (39.2)	268 (63.1)	<0.001
BMI, mean ± SD, kg/m^2^	24.9 ± 3.6	23.7 ± 3.8	0.107
Risk factors, n (%)			
Smoking	52 (32.9)	97 (22.8)	0.013
Hypertension	88 (55.7)	201 (47.3)	0.071
Diabetes	36 (22.8)	84 (19.7)	0.423
COPD	19 (12.0)	38 (8.9)	0.265
Stroke history	9 (5.7)	15 (3.5)	0.242
Heart failure	8 (5.1)	19 (4.5)	0.762
Medications at discharge, n (%)			
Anti-platelets	135 (85.4)	365 (85.9)	0.647
β Blockers	96 (60.8)	246 (57.9)	0.531
RAAS inhibitors	79 (50.0)	203 (47.8)	0.631
Statins	146 (92.4)	379 (89.2)	0.247
Electrocardiographic changes on admission, n (%)			0.708
STEMI	20 (12.7)	49 (11.5)	
NSTEMI	138 (87.3)	376 (88.5)	
Echocardiography, mean ± SD			
LVEF (%)	54.3 ± 13.4	54.9 ± 13.5	0.715
Laboratory parameters on admission, mean ± SD			
Pro-BNP (pg/mL)	986.4 ± 1423.3	919 ± 1356.3	0.778
cTnT (ng/mL)	2.1 ± 1.0	2.0 ± 0.9	0.765
CRP (mg/L)	15.6 ± 4.7	15.7 ± 5.1	0.756
Angiographic data, n (%)			0.760
Normal vessels	18 (11.4)	40 (9.4)	
Stenosis ≤ 30%	72 (45.6)	202 (47.5)	
30% < Stenosis <50%	68 (43.0)	183 (43.1)	

*OSAHS, obstructive sleep apnea-hypopnea syndrome; SD, standard deviation; BMI, body mass index; COPD, chronic obstructive pulmonary disease; RAAS, rennin-angiotensin-aldosterone system; STEMI, ST-segment elevation myocardial infarction; NSTEMI, non-ST-segment elevation myocardial infarction; LVEF, left ventricular ejection fraction; Pro-BNP, pro-brain natriuretic peptide; cTnT, cardiac troponin T; CRP, C reactive protein*.

**Table 2:** The Berlin Questionnaire results and sleep respiratory events recorded by polysomnography.

**Characteristics**	**With OSAHS**	**Without OSAHS**	***P*-value**
	**(*n =* 158)**	**(*n =* 425)**	
AHI, mean ± SD, /h	29.0 ± 12.2	5.3 ± 4.2	<0.001
Baseline SpO2, mean ± SD, %	94.3 ± 4.1	95.0 ± 3.9	0.349
Lowest SpO2, mean ± SD, %	82.7 ± 7.9	91.3 ± 4.1	<0.001
High-risk BQ, n (%)	112 (70.9)	125 (29.4)	<0.001

*OSAHS, obstructive sleep apnea-hypopnea syndrome; AHI, apnea hypopnea index; SD, standard deviation; BQ, Berlin Questionnaire*.

### Medications

Medications prescribed at hospital discharge are presented in Table 1. No significant differences were observed between the OSAHS group and non-OSAHS group in the use of medications at discharge. On the basis of prescription refill at the latest follow-up, 89.4% of the patients in the OSHAS group and 91.2% of the patients in the non-OSAHS group have insisted on antiplatelet therapy. Besides, a total of 89.6 and 87.4% of patients in the OSHAS group were adherent to statin and beta-blocker therapy, compared with 87.9 and 86.8% in the non-OSAHS group, respectively.

### Follow-Up and Clinical Outcomes Medications

**Figure 2:**
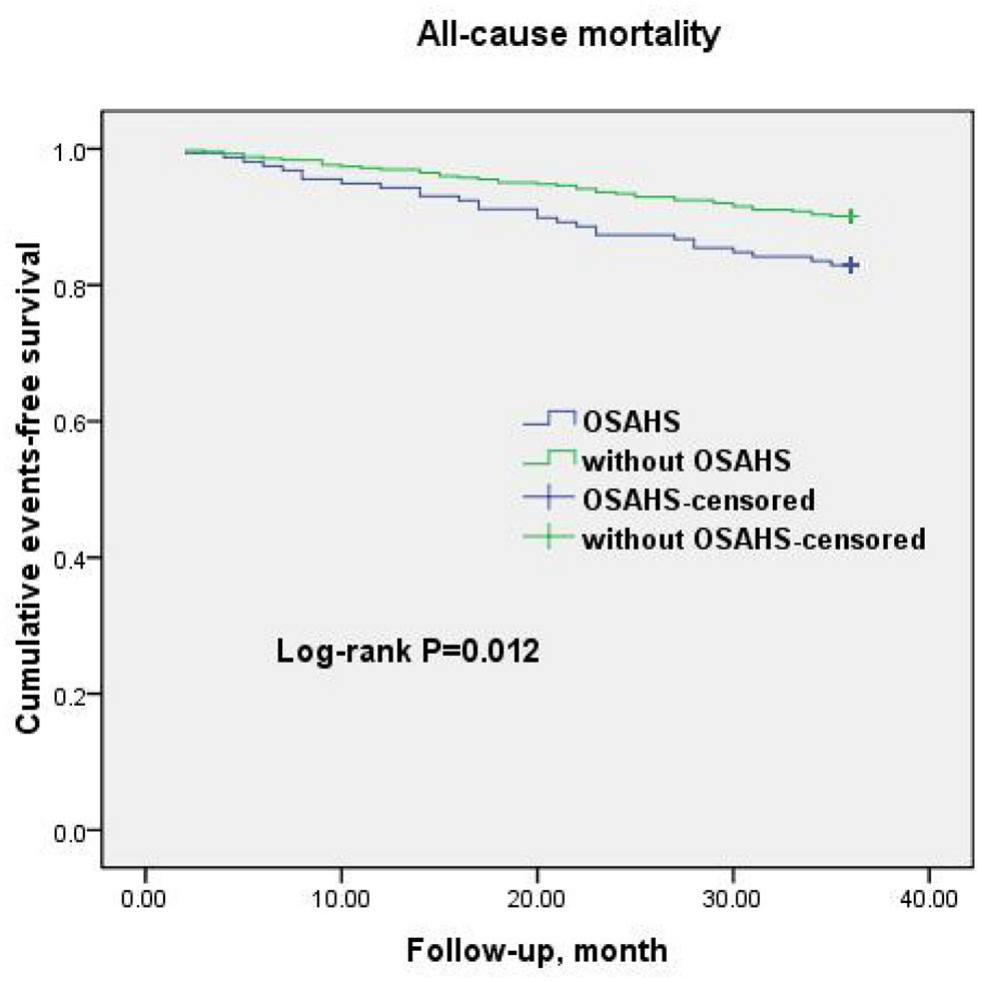
Cumulative event-free survival (Kaplan-Meier curves) for all-cause mortality in OSAHS patients compared to patients without OSAHS.

**Figure 3:**
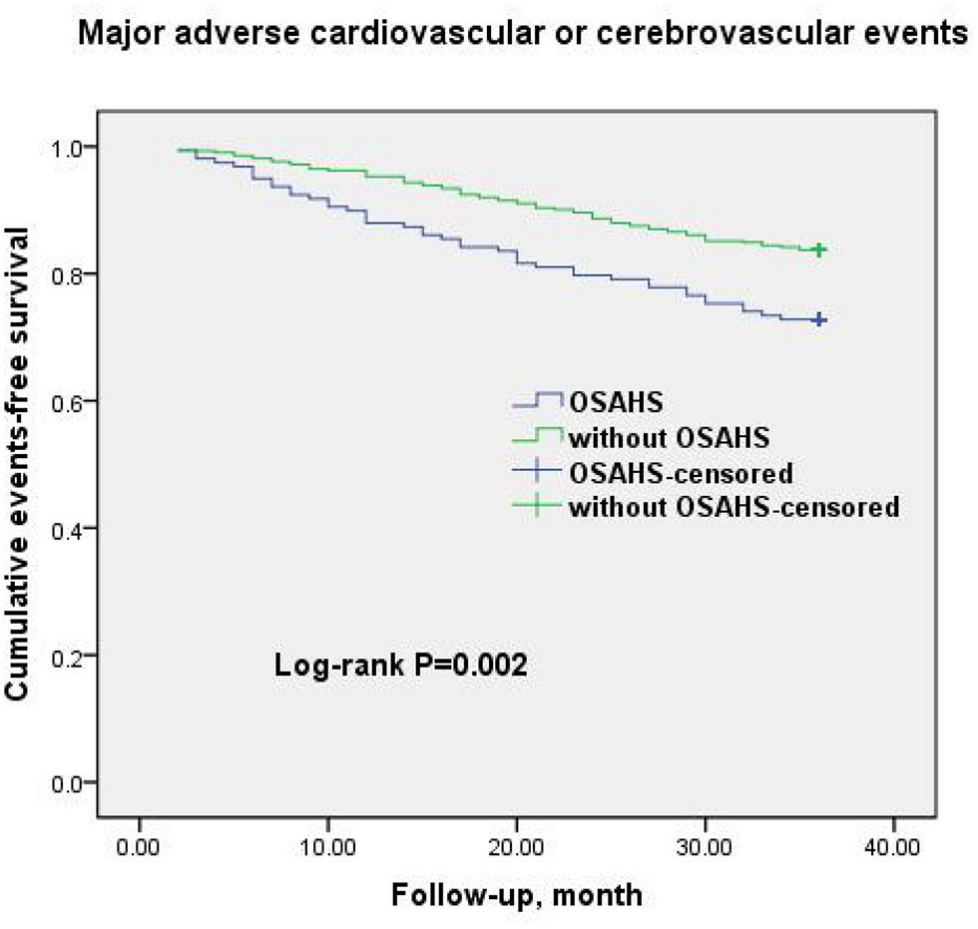
Cumulative event-free survival (Kaplan-Meier curves) for MACCE in OSAHS patients compared to patients without OSAHS.

A total of 69 all-cause mortality and 113 MACCE events occurred during the 3-year observation period in both groups. MACCE included cardiac death in 51 patients (45.1%), non-fatal myocardial infarction in 11 (9.7%), HF in 4 (3.5%), cardiovascular-related rehospitalization in 24 (21.2%), and stroke in 23 (20.6%). The Kaplan–Meier cumulative survival curves indicated a significant association of OSAHS with all-cause mortality (log-rank *P* = 0.012) and MACCE (log-rank *P* = 0.002) (Figures 2, 3, respectively). In univariate Cox regression analysis, OSAHS predicted the incidence of all-cause mortality and MACCE (unadjusted HR = 1.923; 95% CI: 1.411–2.724; *P* < 0.001; and unadjusted HR = 1.977; 95% CI: 1.365–2.856; *P* < 0.001; respectively). After adjustment for age, sex, cardiovascular risk factors, and medications in multivariate Cox regression analysis, OSAHS remained an independent predictor of all-cause mortality and MACCE (adjusted HR = 1.706; 95% CI: 1.286–2.423; *P* = 0.007; and adjusted HR = 1.733; 95% CI: 1.201–2.389; *P* < 0.001; respectively). The results are summarized in Tables 3, 4. Notably, statins, rennin-angiotensin-aldosterone system (RAAS) inhibitors, and ST-segment elevation myocardial infarction (STEMI) revealed a significant correlation with clinical outcomes, agreeing with the findings of previous studies (27).

**Table 3:** Univariate and multivariate Cox regression of variables influencing All-cause mortality.

**Variables**	**Unadjusted HR (95% CI)**	**Adjusted HR (95% CI)**	^*^P-value
Heart failure	1.425 (0.926–1.963)	1.236 (0.884–1.855)	0.112
RAAS inhibitors	0.569 (0.364–0.851)	0.664 (0.390–0.927)	<0.001
Statins	0.601 (0.354–0.670)	0.640 (0.339–0.870)	<0.001
STEMI	3.916 (1.422–9.453)	3.766 (1.416–8.952)	<0.001
OSAHS	1.923 (1.411–2.724)	1.706 (1.286–2.423)	0.008

*HR, hazard ratio; RAAS, rennin-angiotensin-aldosterone system; STEMI, ST-segment elevation myocardial infarction; OSAHS, obstructive sleep apnea-hypopnea syndrome*.

* *The P-value is from multivariate model*.

**Table 4:** Univariate and multivariate Cox regression of variables influencing MACCE.

**Variables**	**Unadjusted HR (95% CI)**	**Adjusted HR (95% CI)**	^*^P-value
Heart failure	1.512 (0.968–2.161)	1.202 (0.807–1.575)	0.077
β-Blockers	0.678 (0.589–0.998)	0.712 (0.675–1.100)	0.042
RAAS inhibitors	0.601 (0.396–0.902)	0.624 (0.412–0.942)	<0.001
Statins	0.466 (0.277–0.746)	0.502(0.297–0.802)	<0.001
STEMI	4.903 (2.142–10.877)	4.633 (1.774–9.680)	<0.001
OSAHS	1.977 (1.365–2.856)	1.733 (1.201–2.389)	<0.001

*MACCE, major adverse cardiovascular or cerebrovascular events; HR, hazard ratio; RAAS, rennin-angiotensin-aldosterone system; STEMI, ST-segment elevation myocardial infarction; OSAHS, obstructive sleep apnea-hypopnea syndrome*.

* *The P-value is from multivariate model*.

## Discussion

To our best knowledge, this research is the first study to explore the prevalence of OSAHS in patients with MINOCA and demonstrate the possible relationship between OSAHS and clinical outcomes in this population. In this cohort of 583 patients with MINOCA, the prevalence of OSAHS was 27.1%, similar to other CAD population. We observed that OSAHS was associated with an increased risk of all-cause mortality and MACCE comprising cardiac death, non-fatal myocardial infarction, HF, cardiovascular-related rehospitalization, and stroke. After adjusting for demographics, cardiovascular risk factors, and discharge medications, patients with OSAHS exhibited 1.706 times risk of all-cause mortality and 1.733 times risk of MACCE at 3-year follow-up. Compared to our earlier study on depression on all-cause mortality and cardiovascular events in patients with MINOCA, the association between OSAHS and clinical outcomes was not that strong (28).

The previous large multi-center registry, The Sleep and Stent Study (11) has shown that obstructive sleep apnea is independently associated with cardiovascular adverse events in patients with CAD, with the prevalence reaching 45.3%. A similar study conducted by Lee also has reported that sleep apnea prevalence is as high as 65.7% in patients admitted with AMI (12). However, the prevalence of OSAHS in patients with MINOCA is unknown. Although the identical diagnostic criterion of OSAHS was an AHI ≥ 15 in the present study, OSAHS was found in 27.1% of patients with MINOCA, which is relatively lower compared with that of CAD patients described by previous studies. In addition, we observed that only 70.9% of the individuals with OSAHS were classified as high risk by the Berlin Questionnaire. The results suggested that the sensitivity and specificity of Berlin Questionnaire remained controversial.

OSAHS is characterized by intermittent upper airway collapse with paradoxical thoracoabdominal movement during sleep, resulting in biochemical and haemodynamic disorders, including sympathetic activation, increased platelet agreeability, hypercoagulability, ultra-inflammation responses, endothelial dysfunction, increased pulmonary vascular resistance, and increased left ventricular load caused by decrease in intrathoracic pressure (27, 29–32). These potential mechanisms together contribute to the progression of atherosclerosis, myocardial infarction, hypertension, arrhythmia, HF, and stroke (8). In the study of Mooe et al. (33) the co-existence of CAD and sleep apnea resulted in 70% relative increase and a 10.7% absolute increase in the composite of death, cerebrovascular events, and myocardial infarction after a median period of 5.1-year follow-up. In one study, obstructive sleep apnea was demonstrated as an independent predictor for clinical outcomes in patients with acute coronary syndrome after PCI (34). The main finding of our observational study is that OSAHS is independently associated with all-cause mortality and MACCE in patients with MINOCA. The total-cause mortality of MINOCA in the 3-year follow-up was 11.8% in the present study, agreeing with the SWEDEHEART registry, with a mortality of 13.4% after mean follow-up of 4.1 years (35). OSAHS patients had an adjusted OR of 1.733 for the MACCE when compared with patients without sleep apnea. Interestingly, we observed that stroke accounted for one-fifth of MACCE in the current study, 16 in patients with OSAHS and 7 in the control group, which is consistent with previous findings indicating OSAHS as an independent risk factor for stroke (13, 36, 37).

The design of present study has certain strengths. First, all patients with OSAHS were diagnosed with gold standard by supervised polysomnography during hospitalization. Second, we rigorously excluded clinically overt causes for a myocardial injury, such as myocarditis, sepsis, pulmonary embolism, Takotsubo syndrome, an potentially overlooked obstructive CAD from the current study, consistent with the clinical algorithm for the recently introduced diagnosis of MINOCA by AHA (4). Therefore, the definition of MINOCA utilized in the current study was consistent with the statement of contemporary diagnosis and management of patients with MINOCA, so the result is more reliable. Third, the well-known confounding risk and preventive factors that affect clinical outcomes in patients with MINOCA, such as traditional cardiovascular risk factors, STEMI, RAAS inhibitors, and statins, were all used for adjustment. Fourthly, the rate of loss to follow-up was low in the present study owing to the convenient instant messaging app (WeChat).

Several limitations need to be considered in the present study. First, the present study is an observational single Chest Pain Center study, and the results may not be generalizable. Second, the sample size of the current study is relatively small due to the critical inclusion and exclusion criteria. Third, the exclusion of patients who refused to participate in the trial and loss of follow-up could contribute to a bias of the results in present study. Finally, one major limitation is that we did not collect longitudinal data on some study covariates prone to change during follow-up period, such as smoking and use of medications after discharge.

## Conclusion

OSAHS is common among patients with MINOCA and is independently associated with increased risk of all-cause mortality and MACCE in patients with MINOCA. The results highlight the importance of management in this population with OSAHS. Intervention and treatment should be considered to alleviate OSAHS-associated risks. Further studies are warranted to determine the effectiveness of CPAP treatment in MINOCA population.

## Data Availability Statement

The raw data supporting the conclusions of this article will be made available by the authors, without undue reservation.

## Ethics Statement

The studies involving human participants were reviewed and approved by Ethics Committee (The First Affiliated Hospital of Jiaxing University). The patients/participants provided their written informed consent to participate in this study.

## Author Contributions

C-JH: conceptualization, methodology, software, investigation, and writing-original draft. L-FC: data curation, formal analysis, and writing- original draft preparation. C-YZ: resources, visualization, and investigation. X-CD: software. Y-JZ: formal analysis, validation, and resources. Y-YY: software and validation. C-LZ: project administration, writing- reviewing, and editing. GQ: validation. H-LH: conceptualization, methodology, writing- reviewing and editing, and supervision. All authors contributed to the article and approved the submitted version.

## Conflict of Interest

The authors declare that the research was conducted in the absence of any commercial or financial relationships that could be construed as a potential conflict of interest.
